# Automated diagnoses of attention deficit hyperactive disorder using magnetic resonance imaging

**DOI:** 10.3389/fnsys.2012.00061

**Published:** 2012-08-30

**Authors:** Ani Eloyan, John Muschelli, Mary Beth Nebel, Han Liu, Fang Han, Tuo Zhao, Anita D. Barber, Suresh Joel, James J. Pekar, Stewart H. Mostofsky, Brian Caffo

**Affiliations:** ^1^Department of Biostatistics, Bloomberg School of Public Health, Johns Hopkins UniversityBaltimore, MD, USA; ^2^F. M. Kirby Research Center for Functional Brain Imaging, Kennedy Krieger InstituteBaltimore, MD, USA; ^3^Department of Radiology, Johns Hopkins School of MedicineBaltimore, MD, USA; ^4^Department of Computer Science, Whiting School of Engineering, Johns Hopkins UniversityBaltimore, MD, USA; ^5^Department of Neurology and Psychiatry, Johns Hopkins School of MedicineBaltimore, MD, USA

**Keywords:** singular value decomposition, random forest, gradient boosting, voxel-based morphometry

## Abstract

Successful automated diagnoses of attention deficit hyperactive disorder (ADHD) using imaging and functional biomarkers would have fundamental consequences on the public health impact of the disease. In this work, we show results on the predictability of ADHD using imaging biomarkers and discuss the scientific and diagnostic impacts of the research. We created a prediction model using the landmark ADHD 200 data set focusing on resting state functional connectivity (rs-fc) and structural brain imaging. We predicted ADHD status and subtype, obtained by behavioral examination, using imaging data, intelligence quotients and other covariates. The novel contributions of this manuscript include a thorough exploration of prediction and image feature extraction methodology on this form of data, including the use of singular value decompositions (SVDs), CUR decompositions, random forest, gradient boosting, bagging, voxel-based morphometry, and support vector machines as well as important insights into the value, and potentially lack thereof, of imaging biomarkers of disease. The key results include the CUR-based decomposition of the rs-fc-fMRI along with gradient boosting and the prediction algorithm based on a motor network parcellation and random forest algorithm. We conjecture that the CUR decomposition is largely diagnosing common population directions of head motion. Of note, a byproduct of this research is a potential automated method for detecting subtle in-scanner motion. The final prediction algorithm, a weighted combination of several algorithms, had an external test set specificity of 94% with sensitivity of 21%. The most promising imaging biomarker was a correlation graph from a motor network parcellation. In summary, we have undertaken a large-scale statistical exploratory prediction exercise on the unique ADHD 200 data set. The exercise produced several potential leads for future scientific exploration of the neurological basis of ADHD.

## 1. Introduction

Attention deficit hyperactive disorder (ADHD) is a highly prevalent psychiatric disorder affecting millions of people. The core symptoms of excessive impulsive, hyperactive, and distractible behavior can have a pervasive impact on functioning across multiple settings with documented long-term consequences including high rates of academic underachievement, unemployment, substance abuse, and criminal activity. ADHD diagnosis currently depends on ratings of behavioral symptoms, which can be unreliable. Better understanding of the physiological, and especially neurological, underpinnings of the behavioral sequelae would be of great use from medical, basic science, and policy perspectives. Moreover, further understanding of the biological basis of the disease would greatly demystify the substantial public uncertainty surrounding the disorder.

ADHD is increasingly recognized as an neurodevelopmental disorder due to converging evidence from structural and functional neuroimaging research. The vast majority of the ADHD neuroimaging literature has focused on pinpointing abnormalities in isolated brain regions [see meta-analyses: Dickstein et al. (2006), Valera et al. (2007)]. However, an emerging etiological model of ADHD has shifted the pathophysiological focus away from localized brain abnormalities toward dysfunctional interactions within and between distributed networks throughout the ADHD brain (Castellanos and Proal, 2011). Recently, correlation patterns in low-frequency, spontaneous blood oxygenation level dependent (BOLD) activity, referred to as resting-state functional connectivity (rs-fc), have been used to characterize the intrinsic functional architecture of the brain [Smith et al. (2009), Biswal et al. (2010)], and a handful of studies have used rs-fc to investigate the neural underpinnings of ADHD [Castellanos et al. (2008), Uddin et al. (2008), Liston et al. (2011), Yang et al. (2011)]. In addition, classifiers have been developed for discrimination of typically developing children and children with ADHD (Zhu et al., 2008). However, considering the heterogeneity of the clinical manifestation of ADHD compared to the sample sizes of most of these studies, the generalization of their findings has been limited.

The ADHD 200 data set is a landmark study compiling over 1000 functional and structural scans including subjects with and without ADHD. As stated on the ADHD 200 website *“Despite advances in understanding aspects of the etiology of some developmental neuropsychiatric disorders, translating these insights into clinical practice has remained daunting. Significant obstacles include the lack of reliable and valid biomarkers and an insufficient understanding of the underlying pathophysiology. We believe that a community-wide effort focused on advancing functional and structural imaging examinations of the developing brain will accelerate the rate at which neuroscience can inform clinical practice.”* Hence, we engaged in the creation of prediction algorithms using ADHD 200 data. Herein we present the insights obtained from the creation of the final ensemble algorithm.

Caution in interpreting the results presented is warranted, as the work was performed while competing in the ADHD 200 prediction competition with the aim of maximizing the competition points earned. The authors of the manuscript include the competitors of the Johns Hopkins team and our collaborators who could not participate in the competition by being members of a data contributing site (Mostofsky, Pekar, Joel and Barber).

The final prediction algorithm presented in this paper had the best official score for predicting the ADHD status of children in the withheld test data. Though we report and discuss the competition results for the full algorithm, we focus on two specific submodels of the final prediction model and evaluate these submodels via diagnostic accuracy using training sample performance rather than external test set performance.

The two primary models of investigation employ feature extraction then ensemble machine learning on the extracted features. The first feature is a voxel selection technique using the so-called CUR decomposition and rs-fMRI. The second evaluates rs-fc regionally in a data-derived motor network mask.

## 2. Materials and methods

### 2.1. Data

The ADHD consortium collected, compiled, and released data from 776 subjects: 491 typically developing controls (TDs) and 285 children diagnosed with ADHD (via standard behavioral symptoms) with subdiagnosis classification of combined, hyperactive/impulsive, or inattentive (American Psychiatric Association, 2000). Each had structural MPRAGE and BOLD functional MRI scans. For numerous subjects, the data were collected over the course of several visits or a few scanning sessions during a single visit. In such cases, features were extracted from each scan separately and averaged within subjects across visits and scanning sessions before inputing into machine learning algorithms. In addition, data from 194 subjects were provided as the testing set to validate competition entries externally. Diagnosis data for many of these subjects has since been released. However, since the selection process of the 194 test set subjects is not known, all measures of algorithmic performance are interpreted with respect to the training sample using data splitting to account for over-fitting.

All models included demographic variables as predictors. These included age, IQ (described further below), gender, and handedness. In addition, data quality control metrics and missing data processes were also investigated. However, these were not used in final algorithms. Available IQ measurement depended on data contributing site and included the WISC IV (Wechsler and Psychological Corporation, 2004), WASI (Weschler, 1999), WISCC-R, two subset WASI, two subset WISC or WAIS Block Design and Vocabulary. The data then included verbal, performance, and two variations of full scale IQ. Our IQ measurement took the median of all available IQ measurements ignoring missingness; we generically label this measurement IQ. All of the other missing values were imputed by using the na.roughfix() command in R. It imputes the missing values of each variable via the median (for quantitative variables) or the mode (for others) for the observed values. All models also included data contributing site, which is a proxy for many processes including technical (scanner, acquisition) and site demographics.

The primary image processing pipeline used the 1000 Functional Connectomes (Biswal et al., 2010) processing scripts available on the NITRC website, and briefly described here (www.nitrc.org/projects/fcon_1000/). Anatomical images were de-obliqued, reoriented, and skull stripped. Functional scans were de-obliqued, reoriented, motion corrected, skull stripped, smoothed (6 mm FWHM Gaussian filter), grand mean scaled, temporal band pass filtered, de-trended (linear and quadratic), and masked to exclude the background voxels (i.e., voxels outside the brain). Functional scans were registered to anatomical scans using FLIRT in FSL (Smith et al., 2004); the structural scans were registered to the MNI 152 (Brett et al., 2004) 3mm T1 template brain using FLIRT and the transformation was subsequently applied to the functional scans. A subset (roughly 50) of functional scans were manually checked for registration performance. Structural scans were then segmented to obtain white matter, and CSF masks. Nuisance regression was performed on functional scans using motion, white matter grand mean, and CSF grand mean. In addition, data from the NeuroBureau's Athena and Dartel pipelines were used. All regional and seed summaries from the Athena pipeline were investigated.

**Figure 1:**
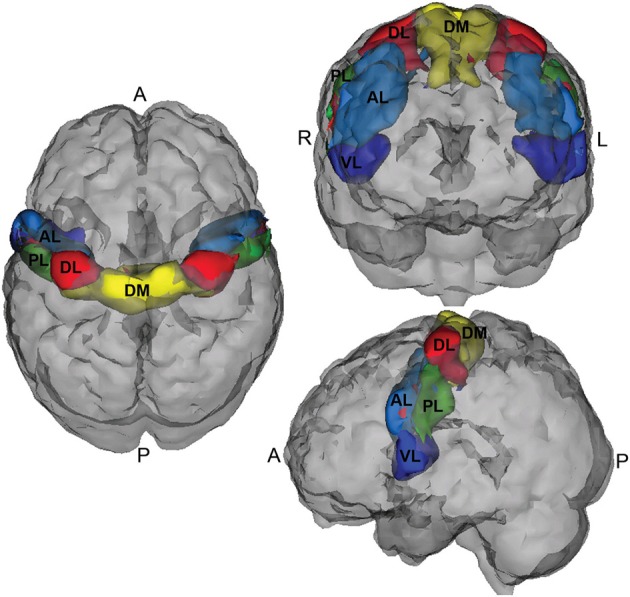
**Motor cortex parcellation**.

A five region parcellation of the motor cortex (M1) (Nebel et al., in press) was used to create connectivity matrices from the NITRC-processed rs-fMRI data. This segmentation was generated using scan–rescan resting state reliability data collected from 20 neurotypical adults (Nebel et al., in press) and reflects the general dorsomedial to ventrolateral organization of the motor homunculus (see Figure 1). This parcellation is fairly right-left symmetric, and its general organization suggests that the dorsomedial parcel (DM, yellow) represents M1 resources involved in control of the trunk/lower limbs; the dorsolateral parcel (DL, red) represent M1 resources dedicated to upper limb control, while the ventrolateral region (VL, dark blue) is involved in oro-motor function.

**Figure 2:**
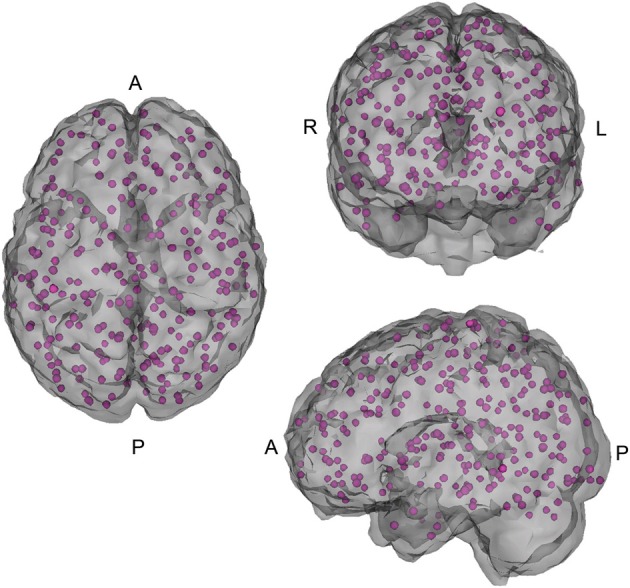
**264 seed voxels**.

In addition, connectivity matrices that broadly cover major functional regions of the cerebral cortex and cerebellum were constructed using 264 reference seeds (see Figure 2) in MNI space (Power et al., 2011b), as well as all of the Athena pipeline seed and regional time courses.

**Figure 3:**
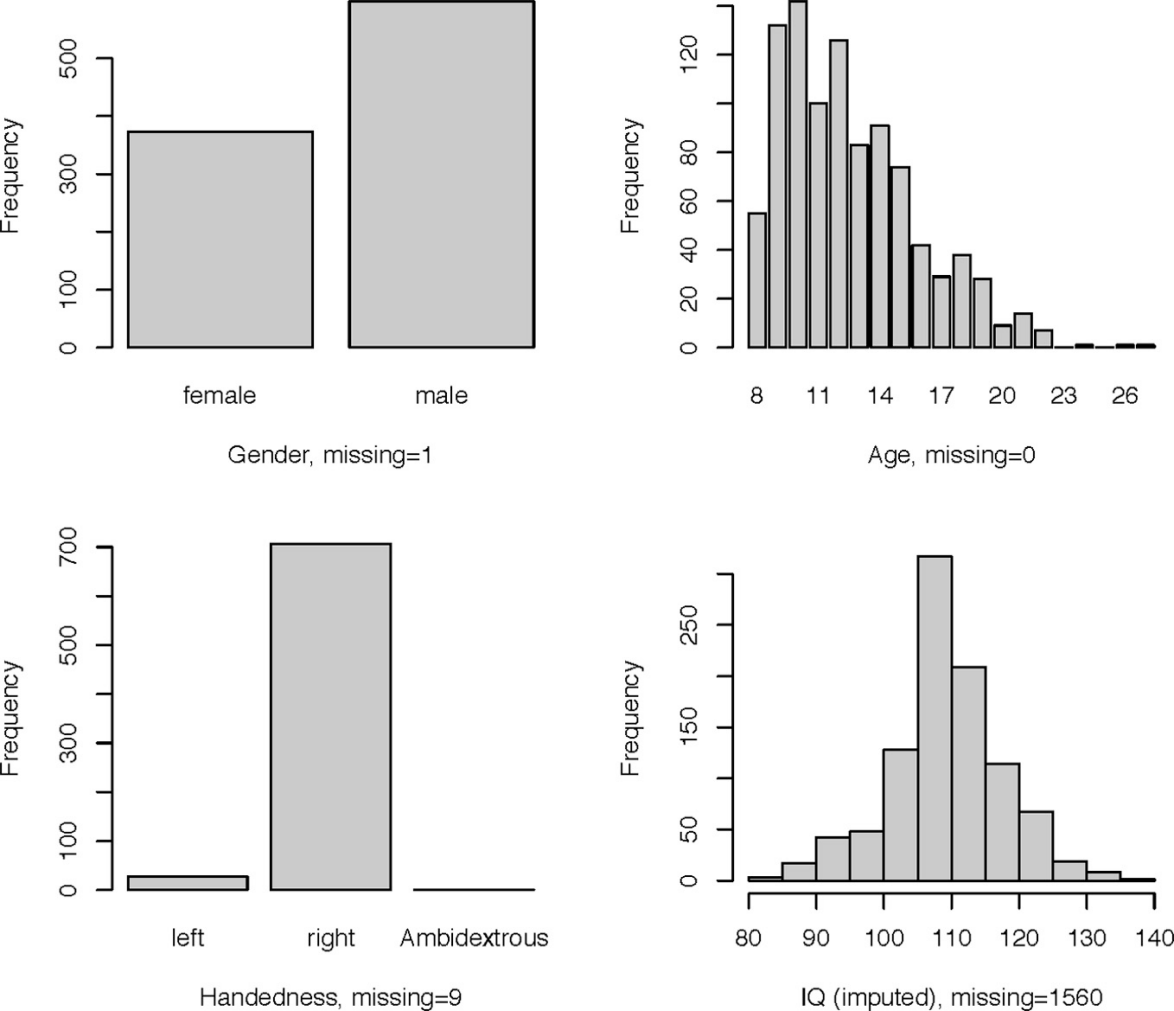
**Demographic information**.

An overview of the demographic information of the children in the study is shown in Figure 3.

### 2.2. Methods

**Figure 4:**
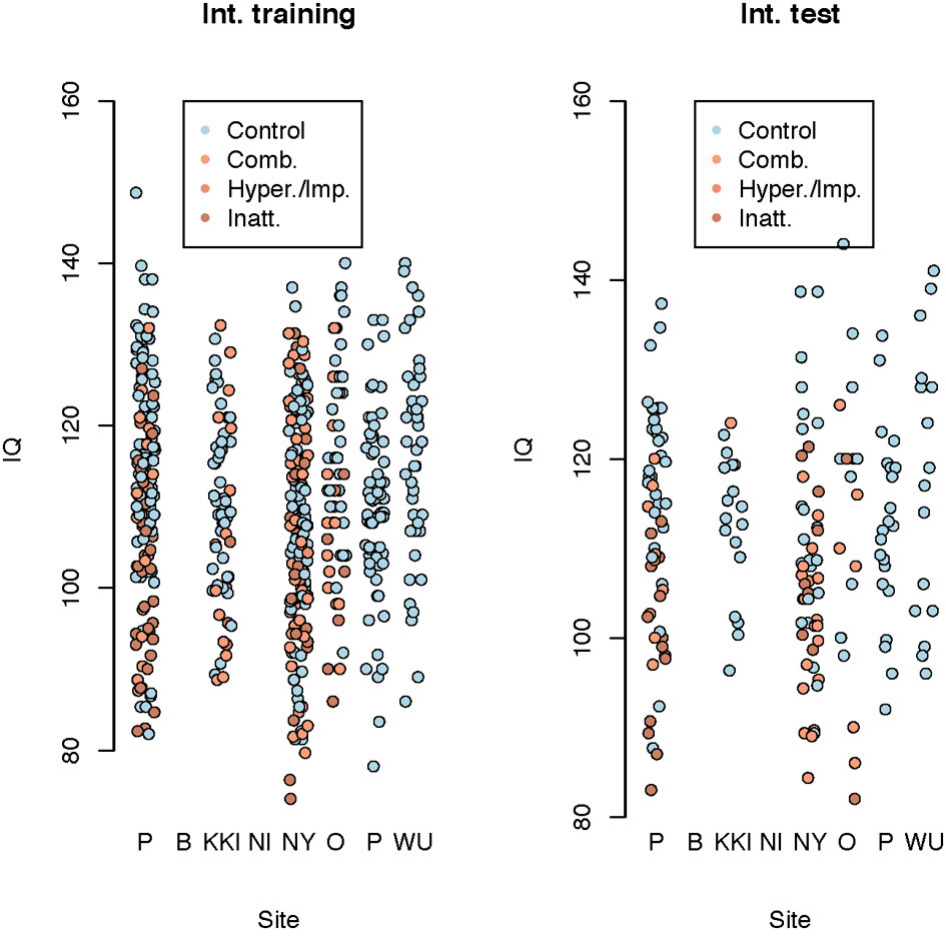
**Dot plot of composite intelligence quotients (average of all available IQ measurements per subject) by data contributing site color coded by disease subtype for the internal training set and internal test set**.

We used several methods—as many as 200 between four subteams—for prediction of ADHD, choosing between the methods via the internal test accuracy measure described below. The methods varied from those using only the covariate data to complex statistical algorithms utilizing the imaging data along with the covariates. We evaluated prediction methods using data splitting where 184 randomly selected subjects were reserved as an internal test set. The internal training set had 363 - TD, 125 - Inattentive, 84 - Hyperactive, and the internal test set had 128 - TD, 38 - Inattentive, 27 - Hyperactive. Figure 4 shows the distributions of the IQ measurements across sites of subjects chosen randomly for internal training and test sets. Algorithms were evaluated by the variant of diagnostic accuracy used in the ADHD 200 competition. A correct classification of a typically developing subject or ADHD subtype yielded one point; classifying a subject as ADHD, but incorrectly classifying subtype yielded 0.5 points. We express total points as a percent of total possible points (which is the sample size, one point per subject). We refer to this measure as “accuracy”; however, we note the distinction from the standard definition of the overall percentage of correct classifications.

The final algorithm was a majority vote of the top algorithm from four subteams (briefly described in Table 1). The top algorithm was chosen to maximize the accuracy as described above from the competition point of view. As classifying an individual correctly as TD earned the algorithm 1 point, this accuracy measure favored algorithms that correctly classified the TD children. The four final “winner” algorithms were combined to obtain a higher accuracy measure. Table 2 shows a brief description of the four methods used in the final prediction algorithm. Briefly, Subteam 1 used random forests for prediction with the 10 (five choose two) correlations of the mean rs-fMRI time courses extracted from the motor network parcellation. For each subject, the average time courses of the 5 parcels were calculated and correlations between the corresponding 10 pairs of the time courses were computed. The 10 correlations for all subjects were stacked and used as predictors in a random forest algorithm along with the other covariates namely age, gender, site, handedness, and IQ measurement. The coefficients of the model were estimated by the random forest (Breiman, 2001) algorithm and then used to predict the ADHD status of subjects in the test set. Subteam 2 used a two step process. First, image features were extracted using online clustering and latent Dirichlet allocation (Blei et al., 2003) based topic models. Here each sample was considered to be one document (collection of words) and the label of each measurement as a word in the vocabulary. K-means clustering (Hartigan and Wong, 1979) was initially applied to the first 10 samples to obtain pilot cluster centers; the clustering structure over the whole data set was then incrementally learned in a stochastic fashion. The extracted image features were combined with the annotation covariates to build predictors using a multi-class support vector machine (Cortes and Vapnik, 1995). Subteam 3 performed a CUR decomposition (Mahoney and Drineas, 2009) on the functional scans along with a gradient boosting method (GBM) [Ridgeway (1999, 2006, 2007) and Freund and Schapire (1995)] for prediction. For each subject, we used CUR decomposition to identify the 20 time courses with the highest variability; we computed the correlations between all pairs (210) of these time courses and applied principal component analysis (PCA) (Jolliffe, 2002) to reduce the number of variables to 10. The resulting 10 variables along with the demographic variables were used in a GBM to predict ADHD status for the test set. Subteam 4 used PCA to reduce the dimension of the pairwise connectivity matrix among the 264 seed voxels. The resulting principal components (reduced matrix), the demographic variables and 36 motion parameters from the Athena pipeline were used in the final model. Gradient boosting was used for prediction in a two-stage fashion, first predicting primary diagnosis (control or ADHD) and then predicting the subtype among those classified as ADHD. Predictions from the four subteams were combined by majority vote to generate the final ensemble prediction, with Subteam 3's prediction used as a tie breaker. The algorithm from Subteam 3 was chosen as the tie breaker since Subteam 3's algorithm had the highest internal test set accuracy.

**Table 1:** **Overview of final prediction methods used by each subteam**.

**Subteam**	**Covariates**	**Processing**	**Methods**
1	All IQ, age, gender, handedness, site	NITRC	Motor network parcellation, random forest random forest for prediction.
2	All IQ, age, gender, handedness, site	NITRC	Feature extraction, clustering, LDA, multi-class SVM.
3	Composite IQ, age, gender, handedness, site	NITRC	CUR decomposition feature extraction, gradient boosting.
4	Composite IQ, age, gender, handedness, site	NITRC NB Athena	264 seed voxels, motion parameters, PCA, machine learning algorithms.

Composite IQ uses the average of all available IQs. All IQ suggests the use of all available IQ measurements. NITRC for image processing implies the use of the 1000 Functional Connectome processing scripts. NB refers to the NeuroBureau pipelines.

**Table 2:** **Basic demographics by site**.

	**Overall**	**Peking**	**Brown**	**KKI**	**NI**	**NYU**	**Oregon**	**Pitt**	**WashU**
**N**	**973**	**245**	**26**	**94**	**73**	**263**	**113**	**98**	**61**
**PERCENTAGE BY SUBTYPE**									
Control	50	47	0	65	32	38	37	91	100
Comb.	17	12	0	17	25	29	20	0	0
Hyper./Imp.	1	0	0	1	8	1	2	0	0
Inatt.	11	20	0	5	1	17	11	0	0
Withheld	20	21	100	12	34	16	30	9	0
**PERCENTAGE BY GENDER**									
Female	38	29	65	40	41	35	46	46	46
Male	62	71	35	60	59	65	54	54	54
**PERCENTAGE BY QUALITY CONTROL**									
% QC Fail	22	1	4	6	12	34	28	32	72
**AGE**									
Min	7.09	8.08	8.50	8.02	11.05	7.17	7.17	10.11	7.09
Median	11.42	11.75	14.83	10.10	17.78	11.11	8.75	14.87	10.35
Mean	12.43	11.70	14.54	10.22	17.64	11.45	9.10	15.08	11.47
Max	26.31	17.33	17.87	12.99	26.31	17.96	12.50	20.45	21.83
Sd	3.33	1.96	2.54	1.34	3.05	2.91	1.25	2.78	3.88

Acronyms are: Comb., ADHD combined type; Hyper./Imp., ADHD hyperactive impulsive; Inatt., ADHD inattentive; % QC fail, percentage where any imaging quality control flag is listed as failing.

**Figure 5:**
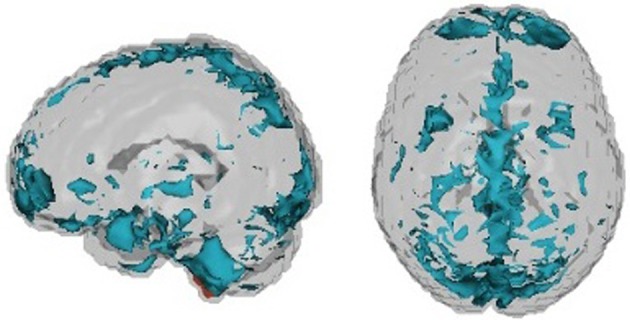
**Voxels chosen by CUR decomposition**.

As it performed well on our internal test data set, we elaborate on the CUR decomposition. We identified the 20 voxels with the highest temporal variability for each subject. The axial and sagittal views of the voxels combined for all subjects are presented in Figure 5. We computed a covariance map for these 20 voxels and, by vectorizing the upper triangle of the covariance matrix, we extracted the covariance vector of the voxels that demonstrated the highest subject-specific variability. Because the number of voxel pairs is still large, we applied singular value decomposition (SVD) to the full covariance matrix to obtain 10 principal components used in the final model. We then fit generalized boosting by combining these 10 principal components obtained from the imaging data with the demographic variables (gender, age, handedness, and combined IQ).

Resting state correlations between the motor network parcels provided the primary avenue of scientific exploration in the data. Therefore, we pursued a more standard analysis of these correlations using multinomial logistic regression with disease status (control, ADHD combined, ADHD inattentive) as the outcome. In addition, we fit a logistic regression model relating ADHD status (regardless of subtype) to rs correlations. Both analyses investigated potential confounding relationships due to demographic factors such as age, data contributing site, etc.

## 3. Results

Table 2 shows basic demographic information for the sample including both withheld and training data. The distribution of the diagnosis varied substantially by site. The sample from Brown University was completely withheld. Training samples from two sites, Pittsburgh and Washington University, were entirely comprised of controls. Sites with more ADHD subjects tended to have a larger majority of males. Failure on any of the quality control metrics varied substantially across sites, presumably due to different data-release policies. Age distributions were similar across sites and ranged between 7 and 26 (years).

**Figure 6:**
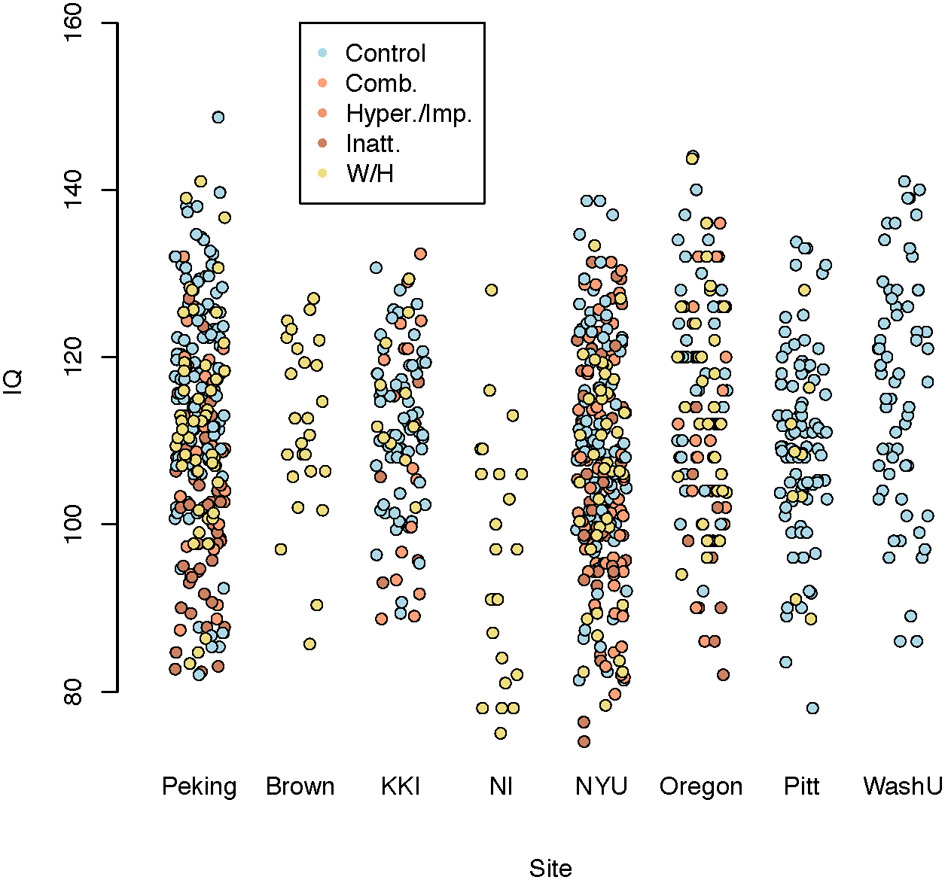
**Dot plot of composite intelligence quotients (average of all available IQ measurements per subject) by data contributing site color coded by disease subtype**.

Figure 6 shows composite IQ measurements by data contributing site. A lower average composite IQ is present for ADHD subjects. The distribution of IQs was consistent across sites, with the exception of Neuroimage, which only provided the two subtest WASI IQ measurements for withheld patients.

With regard to the performance of the final submitted predictions, the internal data-splitting measure of accuracy for each of the subteams was 75, 75, 78, and 72%, respectively. The competition test results can be found at the ADHD 200 web site. The final algorithm test set performance is reported as 119 points (61%). The specificity (control versus ADHD of any type) was reported as 94% with an associated sensitivity of 21%. Youden's J statistic (sensitivity + specificity − 1) was then 15%. The conditional subtype classification accuracy given a correct classification of ADHD was 80%.

We further elaborate on the performance of models from subteams 1 and 3. The internal test set accuracy for the random forest algorithm using only demographic information (age, IQ, gender, handedness, site) was 71% with a specificity of 89% and a sensitivity of 44%. Including the rs correlations from the M1 parcellation resulted in an estimated 75% accuracy with a specificity of 97% and a sensitivity of 35%. For Subteam 3, the two-stage gradient boosting method (GBM) using only demographic variables as input achieved 73% accuracy with a specificity of 71% and a sensitivity of 69%. However, if the CUR imaging decomposition results were included in the model, accuracy improved to 78% with a specificity of 84% and a sensitivity of 53%. These results show that adding imaging information improved the specificity of the algorithms. These findings should be interpreted with caution since the development of the algorithms was focused on improving the prediction accuracy as described in section 2.2, which rewarded the correct classification of TD children.

Table 3 summarizes the results of the mean correlations of pairwise M1 regions by disease subtype and the results of significance tests. Strong inter-subject averages of correlations were found between the posterior lateral (PL) and anterior lateral (AL) parcels (0.450) as well as the VL and PL parcels (0.344). These correlations showed little evidence of differing by subtype. In contrast, the correlations between DM and DL parcels appeared to differ by subtype (*P*-values of <0.01, 0.01 and 0.06 for the three models investigated, respectively), with the lowest correlation among the ADHD combined group.

**Table 3:** **Average fMRI resting state correlations between motor network M1 parcels across subjects classified by disease status subtypes**.

	**VL,DM**	**VL,PL**	**VL,AL**	**VL,DL**	**DM,PL**	**DM,AL**	**DM,DL**	**PL,AL**	**PL,DL**	**AL,DL**
**OVERALL**										
Mean	0.115	0.344	0.183	0.277	−0.002	0.272	0.146	0.450	0.229	0.187
SD	0.206	0.184	0.204	0.191	0.189	0.207	0.201	0.182	0.205	0.187
**CONTROLS**										
Mean	0.134	0.349	0.192	0.284	−0.007	0.279	0.168	0.456	0.241	0.179
SD	0.207	0.182	0.203	0.188	0.183	0.205	0.200	0.174	0.201	0.189
**ADHD COMBINED**										
Mean	0.084	0.349	0.192	0.281	0.008	0.289	0.084	0.469	0.210	0.171
SD	0.209	0.173	0.198	0.185	0.196	0.201	0.194	0.174	0.201	0.198
**ADHD INATTENTIVE**										
Mean	0.103	0.317	0.185	0.249	−0.015	0.266	0.120	0.449	0.239	0.175
SD	0.210	0.183	0.191	0.203	0.187	0.213	0.199	0.187	0.201	0.148
***P*-VALUES TESTING ADHD STATUS BY DISEASE SUBTYPE**										
Model 1	0.023	0.237	0.942	0.212	0.555	0.655	0.000	0.613	0.235	0.884
Model 2	0.440	0.276	0.801	0.241	0.526	0.621	0.012	0.625	0.705	0.925
Model 3	0.418	0.110	0.883	0.657	0.472	0.921	0.057	0.485	0.280	0.701

AL, anterior lateral; DL, dorsolateral; DM, dorsomedial; PL, posterior lateral; VL, ventrolateral. P-values correspond to likelihood ratio tests of multinomial models of the resting state correlation. Model 1 included no covariates, model 2 included gender, age, handedness and IQ, model 3 included model 2 variables plus an indicator for data collecting site.

Given the salience of motor abnormalities in children with ADHD, further exploration of the relationship of intra-motor correlations with disease status is of interest. We used logistic regression where we ignored ADHD subtype, considering only 0 (typically developing) and 1 (ADHD). Each of our 10 models included the four demographic variables along with one pair of M1 clusters as predictors in the logistic regression. First, we found that for most cases, data collection site did not change the direction of the relationship. We found that increased correlation between some pairs of clusters implied significantly lower odds of ADHD, while increased correlation between a few other pairs of motor clusters implied higher (not statistically significant) odds of ADHD.

## 4. Discussion

The ADHD 200 consortium and competition was a remarkable achievement, encouraging scientists from different backgrounds to work collaboratively and competitively on one of the largest collections of (f)MRI data with the goal of advancing our understanding of an important disorder. Our team used hundreds of statistical approaches to predict disease status, and our final ensemble prediction algorithm demonstrated low sensitivity and high specificity. Admittedly, these measures were tuned by the competition rules, which favored methods that correctly identified TD children. Nonetheless, analysis of the results suggests that the imaging data does not provide a great deal of diagnostic benefit, despite several interesting directions of scientific inquiry relating imaging data to disease status being apparent. We elaborate on these points below.

The amount of data provided by the imaging components was very large in the context of statistical prediction. In such cases, if the data has strongly apparent features that are good predictors of outcomes, effective learning procedures can be developed for classification. However, in this case, the amount of data was large enough and the signal weak enough that models were prone to so-called overfitting. In other words, if the imaging predictors are used fully in the model, then predictions may be distorted by the sheer amount of non-informative data.

Gold standard diagnoses were governed by behavioral measures, which themselves are measured with error and are subject to other idiosyncratic biases and variance. Thus, the ultimate goal of the imaging data is to uncover a more accurate phenotype. Perfect agreement with behavioral diagnosis in this data set or others was neither possible nor desirable.

In addition, this data set contained several important sources of variation, some addressable and others not, that influenced our ability to develop meaningful generalizable scientific associations between biomarkers and disease status. A partial list would include: site-specific differences in behavioral measurement, imaging data acquisition, basic processing, scanner quality, technicians and protocols, subject populations from data contributing site including protocols for subject recruitment, policies for contributing data to the consortium, potentially informative missing data processes, as well as other unmeasured confounding and mediating variables. Because of these sources of variation and bias, even weak, non-prognostic associations from this data set may prove invaluable, and conversely, the possibility of identifying spurious associations is quite high. Including site in the regression models improved model performance, suggesting that biologically valueless predictors were, in fact, important.

The demographic makeup of the dataset was also very unbalanced. Data were collected from eight international imaging centers, each with their own research agenda and without coordination prior to aggregation. Thus, many sites collected more data from TD children than from children with ADHD, and this imbalance would cause learning algorithms to err toward classifying subjects as TD as opposed to ADHD. The IQ measurements provided for each subject also varied across sites and resulted in a missing structure that would be predictive of the site where the observation was collected. In developing our algorithms we used imputation methods to account for as much of the data imbalance as possible. For instance, we used the median of all IQ measurements available for each child as a composite measure of IQ in all of our models. However, it is crucial to note that this is an observational study and hence has all of the issues inherent to analysis of imbalanced data.

From our investigations, two approaches used for prediction were especially interesting. One approach appeared to automatically detect residual motion effects that was common across subjects and appeared to differ across diagnostic groups. This raises questions about the residual effect of motion on the statistical analysis and interpretation of fMRI images even after compensatory spatial realignment and regression of motion estimates from the data have been performed. We also observed that a motor network parcellation was a good predictor of disease status.

We pursued the use of the motor parcellation to predict ADHD status based on extensive evidence suggesting that, in parallel to their age-inappropriate impulse control, children with ADHD also demonstrate age-inappropriate motor control. (Denckla and Rudel, 1978) observed that children with ADHD having no learning disabilities show robust patterns of motor overflow consistent with their younger, typically developing counterparts. Motor overflow is defined as unintentional movements that accompany voluntary activity. In a cross-sectional study, (Cole et al., 2008) also showed that, unlike typically developing boys, older boys with ADHD did not show a reduction in motor overflow compared with younger boys with ADHD. Using more quantitative methods involving analysis of video and electrogoniometer data, (MacNeil et al., 2011) showed that children with ADHD exhibit more overflow during a finger tapping task compared to age-matched controls. This sustained motor overflow demonstrated by children with ADHD is thought to reflect immaturity in neural systems involved in unconsciously inhibiting extraneous movement, neural systems that may also be critical for development of behavioral control. (Gilbert et al., 2011) demonstrated that transcranial magnetic stimulation (TMS)-evoked short interval cortical inhibition (SICI) of the M1 was inversely correlated with severity of ADHD; SICI, which may play a role in refining cortical signals involved in selecting motor responses, was reduced by 40% in children with ADHD. In addition, motor skills were evaluated using the Physical and Neurological Examination for Subtle Signs [PANESS, Denckla (1985)], and mean PANESS score was significantly lower for children with ADHD. The combined results from these studies suggests that ADHD may be associated with abnormalities in the connectivity of the motor network.

**Figure 7:**
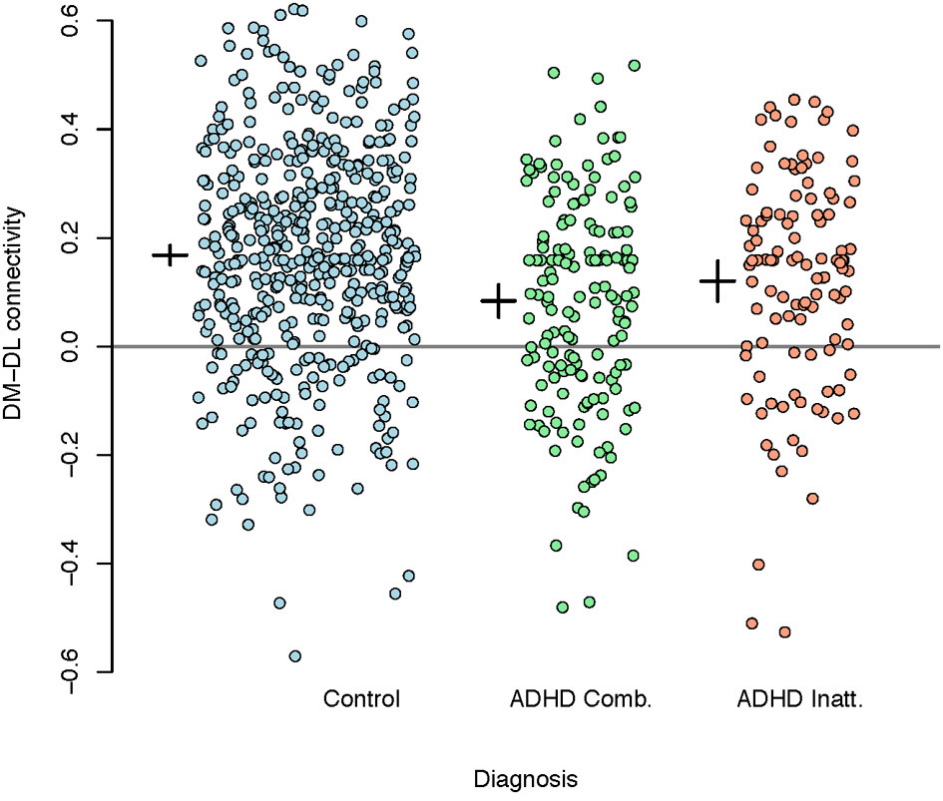
**Plot of correlations between the dorsomedial and dorsolateral M1 parcels by disease subtype.** A reference line is drawn at zero while the inter-subject means (small horizontal line) and 95% confidence intervals (small vertical lines) are given to the left of each group.

Given the extensive discussion in literature on motor control impairments in children with ADHD, we used the M1 parcellation as a predictor of ADHD status. Figure 7 shows the correlations between the DM and DL M1 parcels by disease subtype. We observe that the correlation structure is significantly different for the three disease groups, with combined type ADHD showing the lowest correlation between the DL and DM parcels. However, for all of the reasons outlined above, these connectivity differences may not be very useful for prediction of the ADHD status for an individual subject.

Finally, Table 2 shows the percentage of subjects by site that failed quality assessment tests as given by the organizers of the competition. There is clear variation in quality via either acquisition or choices in what data were shared with the consortium. As argued by (Power et al., 2011a) motion artifacts can have significant effects on correlation-based analyses of resting state fMRI data—even if registration and regression of the motion parameters are performed as a part of preprocessing. This lends credence to the idea that current motion reduction techniques, while removing most of the visible motion, do not capture subtle residual effects of in-scanner motion. Figure 6 shows the voxels identified by the CUR decomposition. (These voxels are a combination map across all subjects.) We observed that the voxels are mostly located in peripheral and CSF regions, suggesting that the CUR decomposition is identifying residual effects of motion. A more thorough discussion of the subject may show the significance of the findings in terms of further reduction of motion-induced artifacts.

In summary, our final prediction models do not provide immediately translatable clinical prediction tools. However, with the collective work of the teams from the competition, numerous interesting directions of scientific inquiry have been uncovered for obtaining a better understanding of the biological basis for this important disorder.

## Funding and acknowledgments

The project was supported by grants P41EB015909 and R01EB012547 from the National Institute of Biomedical Imaging and Bioengineering, grant R01NS060 from the National Institute of Neurological Disorders and Stroke. The organizers of the ADHD 200 competition. The Neurobureau.

### Conflict of interest statement

The authors declare that the research was conducted in the absence of any commercial or financial relationships that could be construed as a potential conflict of interest.
